# Squamous Cell Carcinoma of the Conjunctiva in a Patient With Previous Squamous Cell Carcinoma of the Lower Lip

**DOI:** 10.7759/cureus.33642

**Published:** 2023-01-11

**Authors:** Patricia Chow Liu, Francisca Bragança, Miguel Gomes, Marta Sousa, Katia Ladeira

**Affiliations:** 1 Oncology, Centro Hospitalar de Trás-os-Montes e Alto Douro, Vila Real, PRT; 2 Ophthalmology, Centro Hospitalar Universitário do Porto, Porto, PRT

**Keywords:** ocular surface squamous neoplasia, conjunctival tumor, conjunctiva, conjunctival carcinoma, squamous cell carcinoma (scc)

## Abstract

Squamous cell carcinoma (SCC) of the conjunctiva is a rare malignancy that is part of the spectrum of ocular surface squamous neoplasia (OSSN). Numerous non-modifiable and modifiable risk factors, such as male sex, age, cigarette smoking, and immunosuppression, have been identified. Any lesion of the conjunctiva requires a differential diagnosis between benign and malignant diseases, and all suspicious lesions should be biopsied. We present a case of SCC of the conjunctiva in a former smoker with multiple risk factors, including a previous SCC of the lower lip. Metastatic tumors rarely occur in the conjunctiva, but due to our patient's medical history, the exclusion of metastasis from the previous primary tumor was performed through whole-body imaging restaging. The patient underwent a no-touch wide resection, followed by adjuvant topical chemotherapy with 5-fluorouracil (5-FU). After finishing treatment, the patient continues to attend regular ophthalmology and oncology appointments. Increasing population awareness of modifiable risk factors for OSSN is essential. Misdiagnosis can lead to a loss of time in treatment and progression of the disease.

## Introduction

Ocular surface squamous neoplasia (OSSN) represents lesions of the ocular surface that range from mild epithelial dysplasia to invasive squamous cell carcinoma (SCC) [1,2]. Most arise from conjunctival intra-epithelial neoplasms, although additional precursor lesions include leukoplakia and actinic keratosis of the conjunctiva [1]. There are several known risk factors, including chronic ultraviolet B radiation exposure, cigarette smoking, immunosuppression, and chronic ocular trauma or inflammation [2,3]. Infectious diseases such as human papillomavirus serotype 16 and 18 and HIV type 1 and type 2 infections are also associated with an increased risk of developing OSSN [2]. Recently, the use of topical voriconazole has been proposed as a predisposing factor [4]. Non-modifiable risk factors include male sex and age [2]. SCC has a wide spectrum of presentations and can be confused with several benign and malignant diseases. All suspicious lesions should be biopsied [5]. Standard surgical management includes the traditional no-touch wide resection technique, which consists of removing the tumor with clear margins. After treatment, recurrence is frequent, but regional and distant metastases are extremely uncommon [2,6]. Because of the high recurrence rate with surgical excision alone, surgery is combined with adjuvant therapies such as mitomycin C (MMC), 5-fluorouracil (5-FU), and interferon alpha-2b (INF-α2b) [7]. Topical chemotherapy agents can deliver treatment to the affected surface and the entire ocular surface, potentially eliminating subclinical OSSN [8].

## Case presentation

**Figure 1:**
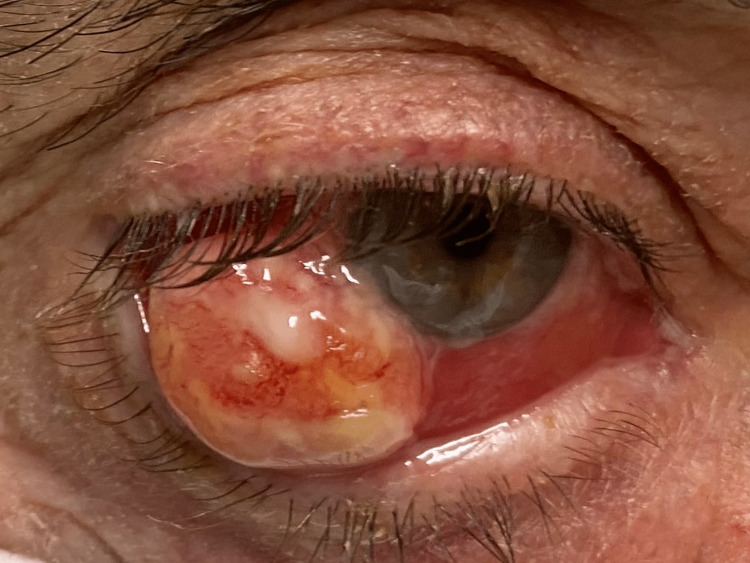
Squamous cell carcinoma of the right eye conjunctiva.

We present the case of a 64-year-old male patient with hypertension, dyslipidemia, type 2 diabetes mellitus, latent syphilis under surveillance, and a former smoker (96 pack years). The patient was under periodic follow-up in oncology consultations due to a local recurrence of an SCC of the lower lip, Tumor, Node, Metastasis (TNM) staging pT2N1M0 of the American Joint Committee on Cancer (AJCC) 8th edition, in January 2021, having undergone surgery followed by adjuvant chemoradiotherapy (total radiation dose of 70 Gy in 35 fractions, five fractions per week, with concomitant cisplatin at a dose of 100 mg/m2 every three weeks on day one, 22, and 43) until May 2021. On August 2021, the patient started to complain of eye irritation and progressive growth on the surface of the right eye (RE) (Figure 1).

**Figure 2:**
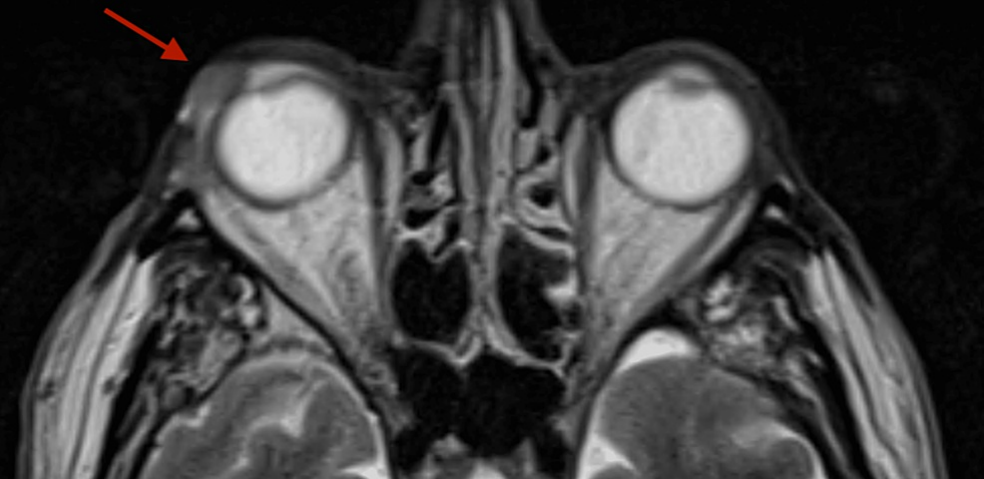
MRI of the brain and orbits showing a lesion in the anterolateral quadrant of the right eye (red arrow).

He was referred to the ophthalmologic consultation, and the biomicroscopy revealed a temporal conjunctival tumor of the RE associated with chemosis and hyperemia without corneal invasion. The histopathologic examination of the tumor biopsy showed a well-differentiated invasive SCC of the conjunctiva. The MRI of the brain and orbits described a lesion capturing contrast in the anterolateral quadrant of the RE (13 mm in greatest anteroposterior diameter and 10.6 mm in greatest transverse diameter) with poorly defined limits, appearing to extend to the upper eyelid and infiltrating the lacrimal gland (Figure 2).

On CT of the face and neck and whole-body positron emission tomography/CT with the glucose analog 2-[18F] fluoro-2-deoxy-d-glucose (FDG-PET/CT), there was no evidence of local or distant recurrence of the SCC of the lower lip.

The patient was referred to the ophthalmology department of a tertiary medical center and underwent a no-touch wide resection of the conjunctival lesion in February 2022. The lesion was located in the inferior temporal quadrant of the bulbar conjunctiva without the involvement of superior or inferior tarsal conjunctiva. Infiltration to the underlying scleral tissue was denoted during the procedure. Cryotherapy was applied to the wound bed and the resection bed's edges. Sponges soaked in MMC 0.02% were then applied to the wound bed and conjunctival edges for three minutes, after which a cryo-preserved human amniotic membrane was sutured, epithelial base-up, to cover the conjunctival defect. The histopathologic examination of the surgical sample confirmed a well-differentiated SCC, with invasion through the conjunctival basement membrane, TNM staging pT2cN0cM0 (AJCC 8th Edition). 

At the Head and Neck Cancer multidisciplinary team meeting, a decision was made to initiate adjuvant treatment with topical chemotherapy. A 5-FU 1% topical solution was applied four times daily in the RE starting in March 2022. After completing two one-week cycles of topical treatment, the patient presented mild ocular surface toxicity with superficial punctate keratitis and two small corneal erosions, which were successfully treated with topical ofloxacin and carbomer, along with the suspension of topical 5-FU for one week. In total, three one-week cycles of topical 5-FU were completed. The decision to finish treatment with 5-FU was made upon the absence of clinical evidence of recurrence. 

**Figure 3:**
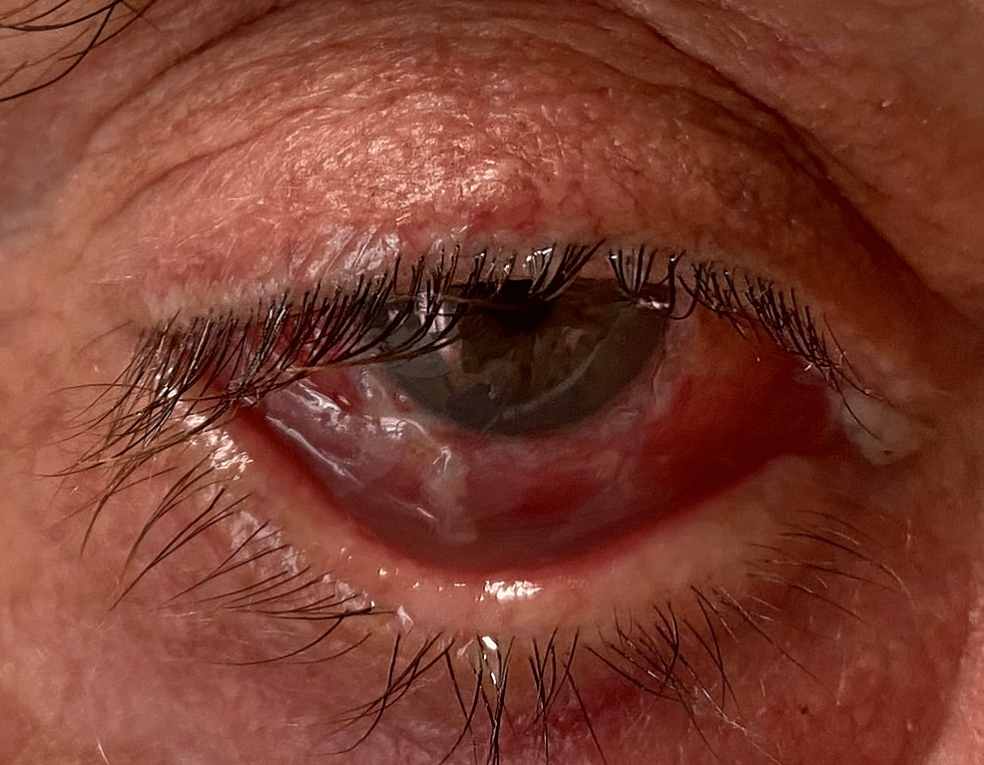
One month after treatment of squamous cell carcinoma of the right eye conjunctiva.

Ophthalmologic consultations were attended weekly during adjuvant topical chemotherapy. On subsequent ophthalmologic consultations until November 2022, there was no evidence of recurrence in the RE (Figure 3). The patient continues to attend regular ophthalmology and oncology appointments.

## Discussion

Invasive SCC of the conjunctiva is a rare malignancy that is part of the spectrum of ocular OSSN [2]. Numerous non-modifiable and modifiable risk factors, such as male sex, age, cigarette smoking, and immunosuppression, have been identified [2,3]. 

The gold standard for diagnosis is histopathologic evaluation following an incisional or excisional biopsy of the conjunctival lesion [2]. The case presented herein illustrates the case of a well-differentiated invasive SCC of the conjunctiva in a former smoker with multiple risk factors, including a previous SCC of the lower lip. 

Metastatic tumors rarely occur in the conjunctiva, but conjunctival metastasis has been described in relation to breast carcinoma, cutaneous melanoma, and other primary tumors [9]. Since our patient had a previous diagnosis of an SCC of the lower lip, exclusion of metastasis from the previous primary tumor was performed through whole-body imaging restaging.

The management of OSSN includes either surgical or medical treatment or a combination of both modalities. The treatment choice depends upon factors such as the patient's age and systemic comorbidities, prior treatments, and ability to comply with medications or to undergo surgery. In patients with documented orbital or intraocular invasion, orbital exenteration or enucleation/radiotherapy may be needed, respectively, and consultation with oculoplastics specialists is required. Standard surgical management includes the traditional no-touch wide resection technique [10], a procedure that, along with intra-operative cryotherapy to the resection bed and edges, aims for clear surgical margins. The use of cryotherapy, leading to tumor cell membrane rupture and blood vessel occlusion, extends the effective surgical margins, and its use has been associated with a reduction in recurrence [7,11]. Adjuvant treatment with drugs such as MMC, as was used in our patient, has been widely used intra-operatively to eliminate residual tumor cells. Even when surgical margins are negative, MMC has been associated with reducing recurrence [9]. Special recommendations for its intra-operative use include recurrent, large, and advanced tumors [12]. Using a cryo-preserved human amniotic membrane graft, as was done in this case, allows for a quicker re-epithelization of the conjunctival defect. An amniotic membrane is the most reasonable option for large tumors whose excision would convey a high risk of limbal stem cell deficiency [12]. 

Histopathologic examination revealed invasion through the conjunctival basement membrane in a well-differentiated SCC, TNM staging pT2cN0cM0 (AJCC 8th Edition). Therefore, a decision was made to proceed to adjuvant topical chemotherapy. Topical chemotherapeutic and immunomodulatory agents, such as 5-FU, MMC, and INF-α2b, are drugs that have been used both as monotherapy and post-operatively in managing OSSN [12]. Its use as adjuvant therapy intends to destroy residual tumor cells [2], mainly in those at high-risk for recurrence. Predictors for OSSN recurrence include positive surgical margins, high histopathologic grade, increased age, and tarsal or scleral involvement, which was verified intra-operatively in our patient [12]. The topical pyrimidine analog 5-FU has been used successfully as monotherapy or in combination with surgical excision in OSSN [2]. Adverse events are usually mild and include conjunctival hyperemia, chemosis, and keratitis, which usually resolve with treatment suspension [12], as in our case. 

## Conclusions

SCC of the conjunctiva is a rare but curable malignancy, and in the presence of a suspicious conjunctival lesion, a very low threshold for biopsy should be maintained to make the diagnosis of SCC at its earliest. Misdiagnosis can lead to a loss of time in treatment and, in the worst case, the progression of the disease to a life-threatening state. Recurrence after the primary excision is frequent, and it is essential to maintain a strict follow-up plan.
